# Telephone Survey as a Method of Data Collection in South India

**DOI:** 10.4103/0970-0218.43237

**Published:** 2008-10

**Authors:** Mahalakshmy Thulasingam, Premarajan K Cheriyath

**Affiliations:** 1 Department of Preventive and Social Medicine, Jawaharlal Institute of Postgraduate Medical Education and Research, Puducherry, India; 2 Department of PSM, Jawaharlal Institute of Postgraduate Medical Education and Research, Puducherry, India

## Introduction

Telephone surveys are popular in developed countries; however, until now this method has not been popular in developing countries because of low telephone coverage.(1) In recent years, with improving telephone coverage, it is a promising method of data collection. It can be used in studies that address an urban population with good telephone coverage. Telephone interviews aid in covering a wider geographical area within less time and with minimal logistics, like transport. The next question will be whether the response to telephone interviews will be good enough to avoid a non response bias. People are more likely to consent for non sensitive issues(2) like child rearing, morbidity, etc. To assess the use of telephone surveys, we conducted a survey on injection practice.

## Materials and Methods

The study was conducted in Kurunji Nagar, an urban area of Puducherry in South India with approximately 600 households. The study involved 50 telephone surveys and 50 face-to-face surveys. For the telephone survey, 100 telephone numbers were randomly selected (using a random number table) from 481 Barath Sanchar Nigam Ltd. (a telecommunication company, BSNL) landline numbers of Kurunji Nagar. Of the 100 telephone numbers, 71 were dialed to cover 50 households. Telephone surveys were conducted in the evenings from 5 p.m. to 7 p.m. on weekdays and from 10 a.m. to 12 p.m. on Sundays, as working men and women are likely to be at home at this time. All surveys were conducted by the same person (female) in November 2006. Any family members older than 18 years old who answered the telephone were interviewed. If the person was younger than 18 years old, he/she was asked to give the phone to a family member older than 18 years.

Initially, the interviewer introduced herself and stated the purpose of the study as following: “A telephone survey is being done to assess the injection practice and preference for injection for treatment of minor illnesses like fever and diarrhea, for which telephone numbers from Kurunji Nagar have been selected and your number is one of them. Kindly respond to a few questions.” However, the probable duration of the survey was not mentioned. They were asked about their preferred route of administration of medicines, knowledge about disease transmission through contaminated syringes, and injection practices in the last 3 months. At the end of the survey, details about the individual were collected. The type of response to the telephone survey was also noted. To check if the results of the telephone survey are similar to that of the face-to-face survey, a sample of 50 houses were selected by multistage sampling. Initially, streets were selected by lots and then houses were selected by systematic random sampling, every third house. To obtain a reliable reply, the head of the family or his wife was asked to answer questions. Face-to-face surveys were also conducted during the same time of the day as the telephone surveys. The amount of time taken for the surveys was also recorded.

## Results

A total of 71 telephone numbers were dialed–8 were nonexistent, 2 were non residential, and there was no response from 8 numbers. Of the 53 houses contacted by telephone, three respondents did not consent for the survey. Of the 52 houses contacted during the face-to-face survey, two respondents did not consent. Five houses were locked and hence 57 houses were visited to cover 50 houses in face-to-face surveys. The response rate of telephone surveys (94.3%) was similar to the face-to-face surveys (96.2%). Of the 50 subjects who consented to participate in the telephone survey, 4 expressed dissatisfaction by interrupting with questions like “How many more questions?” and “What are the benefits of these questions?” Responses of the remaining 46 were good. All subjects who consented completed the survey. The average length of time taken for each questionnaire was 5–6 minutes in both the telephone survey and the face-to-face survey. The amount of time taken for the telephone surveys (5 hours) was half of that taken for the face-to-face surveys (10 ½ hours). All 50 families interviewed in face-to-face surveys had some form of tele-communication facility. Seven families did not have a landline connection but had a mobile phone. All the landlines were BSNL connections.

The survey results are given in Tables 1 and 2. The knowledge and attitude towards the route of administration of medicines were similar in both the survey methods [Table 2].

**Table 1:** Respondent characteristics

	Face-to-face survey (n = 50)	Telephone survey (n = 50)	*P* value
Male:female ratio	1:3.2	1:1.3	< 0.05*
Mean age (range)	40 years (19–62)	42 years (18–73)	0.35
Mean family size	4.3 (1–8)	4.3 (2–8)	1.25
Proportion of graduates and post graduates	62%	54%	0.54

* Significant at *P* less than 0.05

**Table 2:** Attitude and knowledge on route of administration of medicines

	Face-to-face survey n = 50 (%)	Telephone survey n = 50 (%)	*P* value
Prefer oral medication for fever in adults	38 (76)	40 (80)	0.8
Prefer oral medication for diarrhea in adults	42 (84)	44 (88)	0.8
Prefer oral medication for fever in children	40 (80)	39 (78)	1.0
Prefer oral medication for diarrhea in children	40 (80)	40 (80)	1.0
Request for injection in place of oral medicines	9 (18)	6 (12)	0.6
Request for oral medicines in place of injection	16 (32)	12 (24)	0.6
Awareness about transmission of infection through contaminated needles	50 (100)	50 (100)	Not applicable
Awareness about transmission of HIV through contaminated needles	45 (90)	44 (88)	0.7
Awareness about transmission of jaundice through contaminated needles	24 (48)	25 (50)	0.8

## Discussion

Tele-density is the number of telephones per 100 people in a given area; the current tele-density in India is 31% including landlines and mobile phones in urban areas. With the advent of mobile phones, the tele-density (combined urban and rural) is increasing more than expected—from 8.6% in 2004 to 11.4% in 2005. The Department of Telecommunications has set up a target of 22% tele-density by 2007 and is planning to revise the target upward.(3) The improving tele-density allows the option for using telephones as a survey instrument, if the response rate to telephone surveys is good. In this study, the response rate of telephone surveys was similar to that of face-to-face surveys. In Australia,(4) the response rate was around 60% (telephone survey after an introductory postcard) and in the U.S.A., it was 37%.(5) The higher response rate in this study may be due to the short interview time, non sensitive questionnaire, or the rarity of telephone surveys in India. The initial introduction about the interviewer, purpose of the study, and institutional affiliation is important as it improves response rate. Boland M, *et al.* have observed that the question “How many people live in the house at present?” arouses suspicion in some respondents about the threat of burglary(6) but no such observation was noted in this study.

The population covered in the survey was within 1–2 km and the face-to-face surveys took about double the time as compared with telephone surveys. Face-to-face surveys took a longer time for transportation, waiting in case the respondent was involved in some other work, and the respondents wanted to know more about the surveyor and the purpose of the survey. Telephone surveys will be more efficient in saving time in the case of surveys done in a widely distributed area; telephone surveys also allow for sampling from a geographically dispersed area. It not only saves the time of the investigator but also of the respondents. Moreover, working people will be available at home in the evening and they can be contacted by telephone in the evenings. Face-to-face surveys in the evenings are logistically taxing. This also improves the quality of data as informants are more relaxed in the evenings and can spare time for the interview. In telephone surveys, there is saving on time, manpower, and logistics (like transport) and hence it is more economical. Four subjects expressed dissatisfaction; it may be because they were involved in some other work.

The average age and family size were similar in both the methods. There were equal numbers of male and female respondents in telephone surveys comparable to the sex ratio of Puducherry: 1001 females per 1000 males.(7) The telephone surveys selected any person older than 18 years old while the face-to-face surveys selected the head of the household or his wife. Since the interviewer was a female, more often the wife of the head of the family came forward for the interview in the face-to-face surveys. This was the reason for a higher number of females being involved in face-to-face surveys. This bias did not exist in the telephone surveys. The proportion of graduates and post-graduates among males and females were equal in both groups. Knowledge and preference of the route of administration of medicines were similar in both the groups. Knowledge about transmission of diseases by injection was 100% in the present study as compared with 45%, reported from a village in North India.(8) Improved awareness has increased the preference for oral medication. Boland M, *et al.* have commented that anonymity and less social desirability associated with telephone surveys favors the study of sensitive issues like alcohol-related harm.(6) But the response rate for sensitive questions should be studied in the Indian scenario. The survey method has to be further evaluated in other urban and rural areas.

This study included only landlines. Later, during the face-to-face surveys, it was revealed that 14% of the houses selected for face-to-face surveys did not have a landline connection but had a mobile phone. Hence, to ensure representativeness, mobile numbers should also be included in the sampling frame. However, the response rate among mobile phone users has to be evaluated. When contacted over a mobile phone, people may be at their workplace or enroute hence it may not be possible to get reliable information or they may not be cooperative. There may be overrepresentation of houses with multiple mobile phones. A telephone interview is not suitable when the interviewer's observation is also a part of the data collection. Another limitation of the study is that the first adult contact was included and is biased in favor of the person who answers the telephone.

## Conclusion

In this study, the response rate for telephone surveys was good. Characteristics of the subjects and the response in the two survey methods were similar. Hence, telephone surveys are feasible in the Indian scenario and it is a time-saving, useful tool in a population with good telephone coverage. It can be used to rapidly assess availability of services and its utilization and for obtaining feedback on specific programs.
